# Evaluation of the liquid colony for identification and antimicrobial susceptibility testing directly from positive blood cultures

**DOI:** 10.1186/s12941-023-00617-8

**Published:** 2023-08-11

**Authors:** Calvo Maddalena, Migliorisi Giuseppe, Marianna Perez, Scalia Guido, Stefani Stefania

**Affiliations:** 1 U.O.C. Laboratory Analysis Unit, A.O.U. “Policlinico-San Marco”, Via S. Sofia 78, Catania, 95123 Italy; 2 Clinical Microbiology, Department of Biomedical and Biotechnological Sciences, Policlinico Hospital, University of Catania, Via Santa Sofia 97 Third floor, Est tower, Catania, 95124 Italy

**Keywords:** sepsis, Liquid colony, Purified microbial suspension, Time-saving workflow

## Abstract

**Background:**

Sepsis represents a time-sensitive disease requiring early therapeutical intervention to avoid adverse patient outcomes. Rapid microbiological diagnosis is essential to investigate sepsis aetiological agents. The FAST™ system (Qvella, ON, Canada) provides a concentrated microbial suspension, known as a Liquid Colony™ (LC), directly from positive blood samples (PBCs) in 30–40 min to perform rapid identification (ID) and antimicrobial susceptibility testing (AST).

**Methods:**

Qvella’s FAST™ System and FAST PBC Prep cartridges were tested on PBCs from the Policlinico Hospital of Catania during a six-month study. Two millilitres of PBC were converted into an LC for rapid ID and AST using Bruker Biotyper Sirius MALDI and BD Phoenix systems. Standard of care (SOC) methods were used as a reference, requiring 48–72 h. Agreement between the innovative technology and the standard method was calculated.

**Results:**

FAST System processing was performed on 100 monomicrobial PBCs. Median turnaround times from blood cultures flagging positive to ID and AST completion were 2 and 26 h respectively. Therefore, the LC procedure was 24 h faster than the median turnaround times for SOC methods. 100% ID identification concordance was observed across 48 Gram-negative bacteria, 42 Gram-positive bacteria and 11 yeast for the genus level. 78% of Gram-negative and 95% of Gram-positive bacteria were resistant to ≥ 2 antimicrobial agents, including 45% (15/33) carbapenem-resistant enteric Gram-negative bacteria and 90% (28/31) oxacillin-resistant staphylococci. An AST essential agreement of 100% was observed due to the absence of MIC discrepancies > 1-fold dilution. Categorical errors were not observed due to the absence of MIC categorization discordances. Yeast AST was not performed.

**Conclusions:**

The Qvella FAST System produces an LC that reliably reflects the MALDI spectra and phenotypic antimicrobial susceptibility profile of microbial cells growing in the blood culture. Timely processing of PBCs with the Qvella FAST System enables sepsis diagnostic confirmation 1 day sooner than the standard methods. In line with these results, it is vital now to focus attention on establishing best practices for incorporating this strategic tool into the clinical microbiology laboratory workflow.

## Introduction

Sepsis is one of the most time-dependent life-threatening conditions, caused by a dysregulated host response to severe infectious diseases such as bacterial or fungal bloodstream infection, or respiratory tract infection with systemic complications. The latest World Health Organization (WHO) analysis reported a global sepsis rate of 49 million individuals. These data include approximately 11 million potentially avoidable deaths [1, 2]. Late diagnosis is the leading cause of sepsis mortality worldwide [1, 3].

The current standardized clinical microbiology diagnostic procedures for bloodstream infection begin from a flagged positive blood culture, which subsequently undergoes an overnight subculture before identification (ID) and antimicrobial susceptibility testing (AST). Including time for growth of colonies on agar plates, ID and AST often require 48–72 h until results are available. These protracted procedures are mandatory to complete a standardized diagnostic workflow and are dependent on microbial growth rates.

Pending a microbiological diagnosis, clinicians administer empiric antimicrobial therapy for sepsis to prevent severe clinical complications or death in affected patients. The turnaround time for blood culture results becomes critical to achieve early information about sepsis aetiological agents and optimal antimicrobial therapy. Meanwhile, hesitations in placing targeted therapy can often lead to a negative outcome due to time dependence on sepsis clinical progress. The urgency to treat with broad spectrum empiric therapy often leads to unnecessary antimicrobial exposure, which have recently increased as noted by the Center for Disease Control (CDC) [4].

Rapid identification of sepsis aetiological agents and their susceptibility profiles enables clinicians to provide appropriate targeted antimicrobial treatment. A number of diagnostic tools claim to provide rapid molecular ID or AST data in minutes or a few hours, directly starting from a positive blood culture. However, phenotypic AST confirmation is often needed through conventional culture-based methods, excepting some validated standard-of-care fast procedures [5–10].

This study aimed to evaluate the Qvella FAST™ System and FAST PBC Prep Cartridge for the production of a purified and concentrated microbial suspension, or Liquid Colony™ (LC), directly from a positive blood culture, and the performance of the LC for rapid ID and AST protocols. Standard culture-based methods were performed in parallel as the reference method to produce a comparative analysis.

## Methods

The study was performed from July to November 2022 at the University Hospital Policlinico of the University of Catania. Positive blood cultures (PBCs) were prospectively included from patients recovered in intensive care, haematology, internal medicine, and emergency units. An experimental protocol was approved by the University of Catania Institutional Review Board to compare the Qvella FAST™ System to the standard for clinical microbiology diagnostic methods.

Briefly, the standard method consisted of a Gram-stain preparation from a PBC flagged as positive after incubation in the Beckton Dickinson BD BACTEC™ FX System (Plus Aerobic medium, Plus Anaerobic medium, Peds Plus medium and Mycosis). That step was crucial to exclude polymicrobial blood samples from the study. After the Gram-stain results were transferred to clinicians in a timely way, a portion of the positive sample underwent standard procedures [11]. Colonies obtained by subculture on solid agar were identified using the Matrix-Assisted Laser Desorption/Ionization Time-of-Flight (MALDI-TOF) Biotyper® Sirius System (Bruker Daltonics) as described below. Bacterial isolates were also placed into BD Phoenix™, an automated ID and AST system providing a definitive report (see below for details). NID (identification panel) and NMIC-474 (AST panel) were used for Gram-negative bacteria, while PMIC/ID-88 (identification and AST panel) was used for Gram-positive bacteria.

After the Gram stain, the rapid FAST System (Qvella Corporation, Richmond Hill, ON, Canada) protocol was performed in parallel as follows: 2 ml of each PBC sample was inoculated into a single-use FAST PBC Prep cartridge and run for 30–40 min using the automated FAST System instrument. The result was a purified, concentrated suspension of microbial cells called LC. The automated PBC system required only 1 to 2 min of hands-on time by laboratory personnel. 1 µl of the LC was used for the ID process by MALDI-TOF as described in the following paragraph.

The LC and colonies from subcultures on solid agar were spotted on a steel multi-use MALDI MBT Biotargets 96 and 1 µl of an *in-vitro* diagnostic (IVD) α-Cyano-4-hydroxycinnamic acid (HCCA) Matrix was added. Formic acid (70%) was added before the matrix addition only in case of suspected Gram-positive bacteria or yeast derived from the initial Gram-stain. MBT Compass IVD 4.2 was used to perform identification. IVD bacterial test was acquired during all procedures to provide process control. According to the solid colonies protocol, a 2.0 score was accepted for correct identification. Scores from 1.7 to 1.99 were also accepted, but they underwent a remeasurement to improve identification spectra. However, these values were considered reliable identification only to the Genus level.

Suspensions of colonies from subculture on solid agar and the remainder of the LC was used to prepare a 0.5 MacFarland inoculum for BD PhoenixTM ID and AST panel, which functions based on turbidity and colourimetric principles. Manual Kirby-Bauer and Gradient test methods were applied to liquid and solid colonies in the case of uncommon organisms, whose susceptibility profile was not included in the automated system. All AST profiles were evaluated according to EUCAST definition guidelines [12]. Categorical agreement (CA), essential agreement (EA), and minor, major, and very major error rates were calculated according to standard guidelines [13]. AST was not performed from LCs obtained from samples containing yeast.

**Graph. 1:**
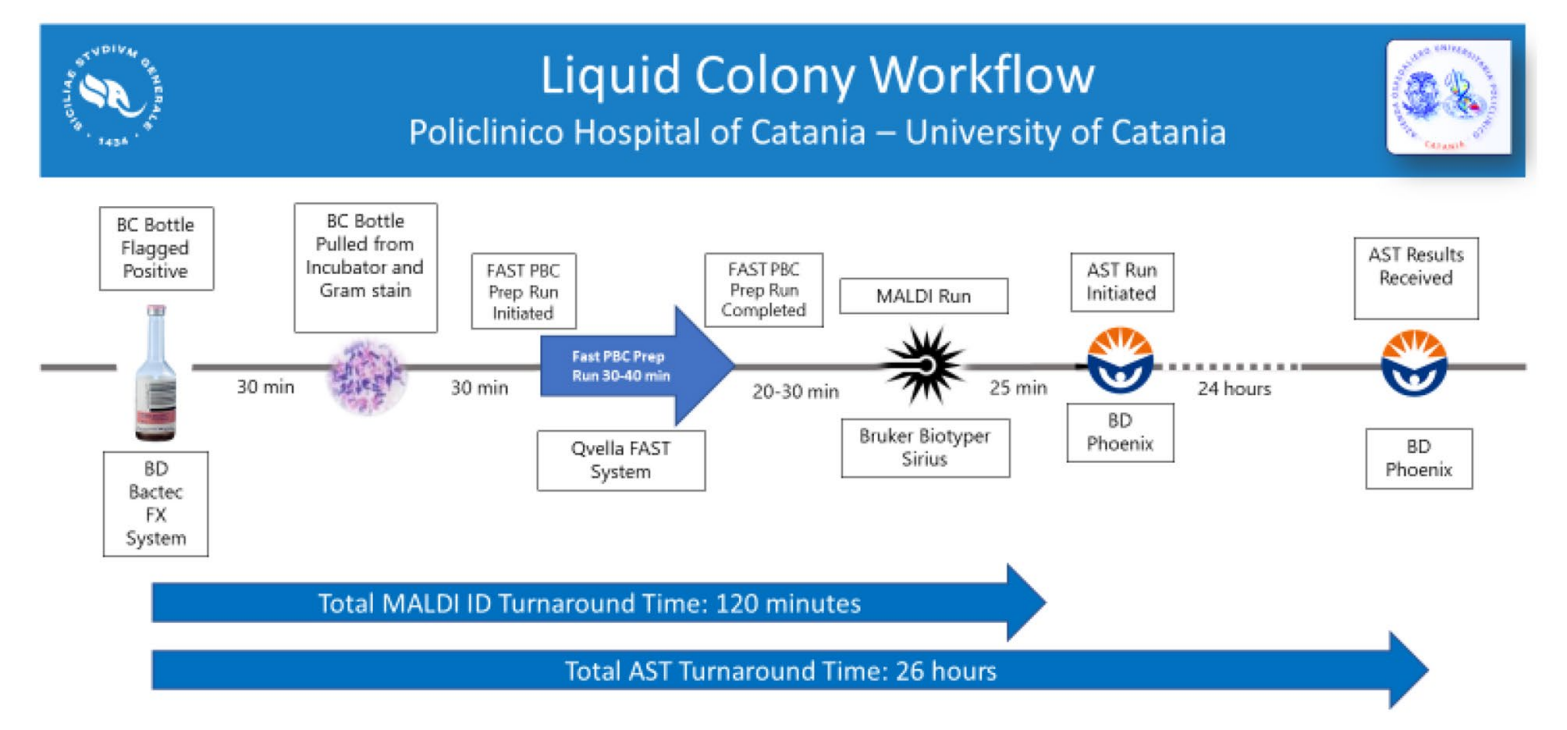
**Schematic illustration of Liquid Colony workflow for positive blood cultures**. Shown are median times from blood culture (BC) bottle flagged positive to BC bottle pulled from the incubator, to initiation of FAST PBC Prep run, to initiation of antimicrobial susceptibility testing (AST), to MALDI identification (ID), to AST completion

An overview of the liquid colony workflow is illustrated in Graph 1.

## Results

### Identification

The study globally collected 86 positive blood cultures from adult patients and 14 from paediatrics. As shown in Tables 1 and 47 samples tested positive for Gram-negative bacteria, 42 showed Gram-positive bacteria, and 11 samples revealed yeast. Specifically, 33 isolates belonged to the *Enterobacterales* (20 *Klebsiella pneumoniae* subspecies *pneumoniae*, 5 *Escherichia coli*, 3 *Enterobacter cloacae* complex, 1 *Enterobacter aerogenes*, 2 *Proteus mirabilis*, 2 *Serratia marcescens*). Furthermore, non-fermentative Gram-negative rods were confirmed to be *Acinetobacter baumannii* (8), *Acinetobacter berezinae* (1), and *Pseudomonas aeruginosa* (4). Uncommon identification of *Capnocytophaga sputigena* (1) was also performed. Specifically, 3 microorganisms (1 *E. aerogenes*, 1 *K. pneumoniae* and 1 *Enterobacter cloacae* complex) were identified through a MALDI score lower than 2.0, confirming only a genus identification.

Regarding Gram-positive bacteria, 6 *Enterococcus faecium* and 2 *Streptococcus* species (1 *Streptococcus oralis* and 1 *Streptococcus mitis*) were detected. Moreover, 31 isolates were attributable to the *Staphylococcus* genus (9 *Staphylococcus aureus*, 14 *Staphylococcus haemolyticus*, 7 *Staphylococcus epidermidis*, and 1 *Staphylococcus hominis*). Finally, 1 *Bacillus cereus* and 1 *Clostridium paraputrificum* were also recognized. Thirteen microorganisms (1 *C. striatum*, 1 *S. aureus*, 5 *S. epidermidis*, 3 *S. haemolyticus*, 1 *C. paraputrificum*, 1 *E. faecium* and 1 *S. mitis*) were identified after a MALDI score lower than 2.0, confirming only a genus identification. A medium time to positivity of 16 h was reported both for Gram-negatives and Gram-positives. Gram-negatives required a minimum time to positivity of 2 h and a maximum of 116 h. Otherwise, Gram-positives required from 5 to 49 h. 1 sample tested positive (19 h) for *Corynebacterium striatum*.

11 samples revealed yeast isolates. Notably, 10 strains were attributable to the *Candida* genus (6 *Candida parapsilosis*, 2 *Candida tropicalis*, and 2 *Candida albicans*). Only 1 strain was identifiable as *Magnusiomyces capitatus*. A medium time to blood culture positivity was recorded as 22 h, with a minimum time to positivity of 15 h and a maximum to positivity of 65 h. All the yeast identifications revealed a MALDI score lower than 2.0, allowing only a genus confirmation.

**Table 1:** Species identified in positive blood samples

Gram-negative bacteria	Isolates (#)
*Acinetobacter baumannii*	9
*Acinetobacter berezinae*	1
*Capnocytophaga sputigena*	1
*Enterobacter aerogenes*	1
*Enterobacter cloacae*	3
*Escherichia coli*	5
*Klebsiella pneumoniae*	20
*Proteus mirabilis*	2
*Pseudomonas aeruginosa*	4
*Serratia marcescens*	2
Total	48
Gram-positive bacteria	Isolates (#)
*Bacillus cereus*	1
*Clostridium paraputrificum*	1
*Corynebacterium striatum*	1
*Enterococcus faecium*	6
*Staphylococcus aureus*	9
*Staphylococcus epidermidis*	7
*Staphylococcus haemolyticus*	14
*Staphylococcus hominis*	1
*Streptococcus mitis*	1
*Streptococcus oralis*	1
Total	42
Yeast	Isolates (#)
*Candida albicans*	2
*Candida parapsilosis*	6
*Candida tropicalis*	2
*Magnusiomyces capitatus*	1
Total	11

There was 100% genus-level concordance between ID from LCs obtained using the Qvella FAST System and ID from colonies obtained from solid agar subcultures using MALDI-ToF. The average MALDI ID scores were in line with the manufacturer’s guidelines, which categorize as reliable species identifications all the scores ≥ 2.0 (green signal) and reliable genus identifications all the scores between 1.70 and 1.99 (yellow signal).

27% of the identified isolates revealed a MALDI score lower than 2.0. Specifically, 5 *S. epidermidis* reported scores between 1.74 and 1.94, while 3 *S. haemolyticus* revealed scores between 1.94 and 1.98. 1 *E. faecium* registered a score equal to 1.92 and 1 *S. aureus* reported a value of 1.74. 1 *C. paraputrificum* reported a score of 1.80, while 1 *S. mitis* and 1 *C. striatum* respectively recorded score values of 1.98 and 1.87. 1 *E. aerogenes* reported a score value of 1.94, while 1 *E. cloacae* and 1 *K. pneumoniae* respectively revealed scores of 1.80 and 1.98. 1 *M. capitatus* revealed a score value of 1.94, while 2 *C. tropicalis* reached a range of 1.70–1.98. A number of 2 *C. albicans* reported a score range of 1.74–1.98. Finally, 6 *C. parapsilosis* reported a range of 1.74–1.98.

### Antimicrobial susceptibility testing

Gram-negative bacteria were tested with 24 different antibiotics and resistance was noticed in 53% of cases (Table 2). Gram-positive bacteria were tested with 23 different antibiotics and resistance was noticed in 36% of cases. (Table 3). 78% of Gram-negative bacteria including were resistant to ≥ 2 antimicrobial agents, including 45% (15/33) of enteric Gram-negative bacteria resistant to carbapenems. 95% of Gram-positive bacteria were resistant to ≥ 2 antimicrobial agents, and 90% (28/31) of staphylococci were oxacillin-resistant.

Uncommon strains, whose susceptibility profiles were not included in the automated systems, were tested through the Gradient test and Kirby-Bauer techniques. Specifically, ciprofloxacin, clindamycin, linezolid, vancomycin and rifampicin were tested for *C. striatum*, while vancomycin, clindamycin, metronidazole and meropenem were applied to *C. paraputrificum*. Furthermore, *B. cereus* was tested with erythromycin, clindamycin, linezolid, vancomycin, ciprofloxacin, levofloxacin, meropenem and imipenem. Finally, ampicillin/sulbactam, cefotaxime, imipenem, meropenem, ciprofloxacin, levofloxacin and trimethoprim/sulfamethoxazole were tested on *C. sputigena*.

**Table 2:** Antimicrobial susceptibility rates for Gram-negative bacteria

Gram-negative bacteria	S	I	R	%S
Amikacin	28	0	17	62%
Ampicillin/sulbactam	1	0	0	0%
Amoxacillin-clavulanic acid	5	0	28	15%
Ceftazidime-avibactam	32	0	5	86%
Ceftolozane-tazobactam	22	0	15	59%
Ciprofloxacin	16	5	17	45%
Gentamicin	20	0	23	47%
Imipenem	26	0	18	58%
Levofloxacin	17	0	27	73%
Meropenem	30	0	18	62%
Meropenem-vaborbactam	33	0	0	100%
Piperacillin-tazobactam	10	0	27	27%
Trimethoprim-sulfamethoxazole	8	0	36	82%
Tobramycin	19	0	28	40%
Temocillin	0	13	17	0%
Ampicillin	5	0	28	15%
Aztreonam	12	0	21	36%
Cefepime	12	5	20	32%
Cefotaxime	1	0	0	0%
Ceftazidime	12	4	21	32%
Ceftriaxone	12	0	21	36%
Cefuroxime	7	0	23	23%
Ertapenem	18	0	15	55%
Colistin	0	0	2	0%
Totals	346	27	427	43%

**Table 3:** Antimicrobial susceptibility rates for Gram-positive bacteria

Gram-positive bacteria	S	I	R	%S
Ceftaroline	31	0	0	100%
Ciprofloxacin	6	0	29	17%
Levofloxacina	1	0	0	100%
Clindamycin	31	0	6	84%
Daptomycin	31	0	0	100%
Erythromycin	6	0	26	17%
Gentamicin	15	0	23	39%
Linezolid	36	0	5	88%
Moxifloxacin	3	0	28	10%
Mupirocin	31	0	0	100%
Oxacillin	3	0	28	10%
Fusidic Acid	31	0	0	100%
Penicillin G	0	0	31	0%
Teicoplanin	39	0	0	100%
Tetracycline	30	1	0	97%
Tigecycline	31	0	0	100%
Trimethoprim-sulfamethoxazole	8	0	23	26%
Ampicillin	0	0	7	0%
Imipenem	1	0	7	12,5%
Rifampicin	0	0	2	0%
Vancomycin	44	0	0	100%
Meropenem	2	0	0	100%
Metronidazole	1	0	0	100%
Totals	381	1	215	64%

Despite the high percentage of multidrug-resistant bacteria, few AST discordances were observed between the LC and suspensions of colonies obtained from solid agar subculture. Four 1-fold dilution differences in minimal inhibitor concentration (MIC) were observed, though none of these differences resulted in categorical errors. No MIC discordances > 1-fold dilution were observed. Specifically, 2 *S. haemolyticus* liquid colony reported a MIC value of 0.5 mg/L, whose corresponding solid colony value was 0.25 mg/L for daptomycin. 1 *S. haemolyticus* revealed a liquid colony MIC value of 2 mg/L, whose corresponding solid colony value was 1 mg/L for linezolid. Finally, 1 *K. pneumoniae* respectively reported 0.25 mg/L and 0.5 mg/L for liquid and solid colonies testing about ciprofloxacin.

As a result, 100% categorical and essential agreement were recorded for both Gram-positive and Gram-negative bacteria. No minor, major, or very major errors were observed.

### Turnaround Time (TAT) analysis

**Graph. 2:**
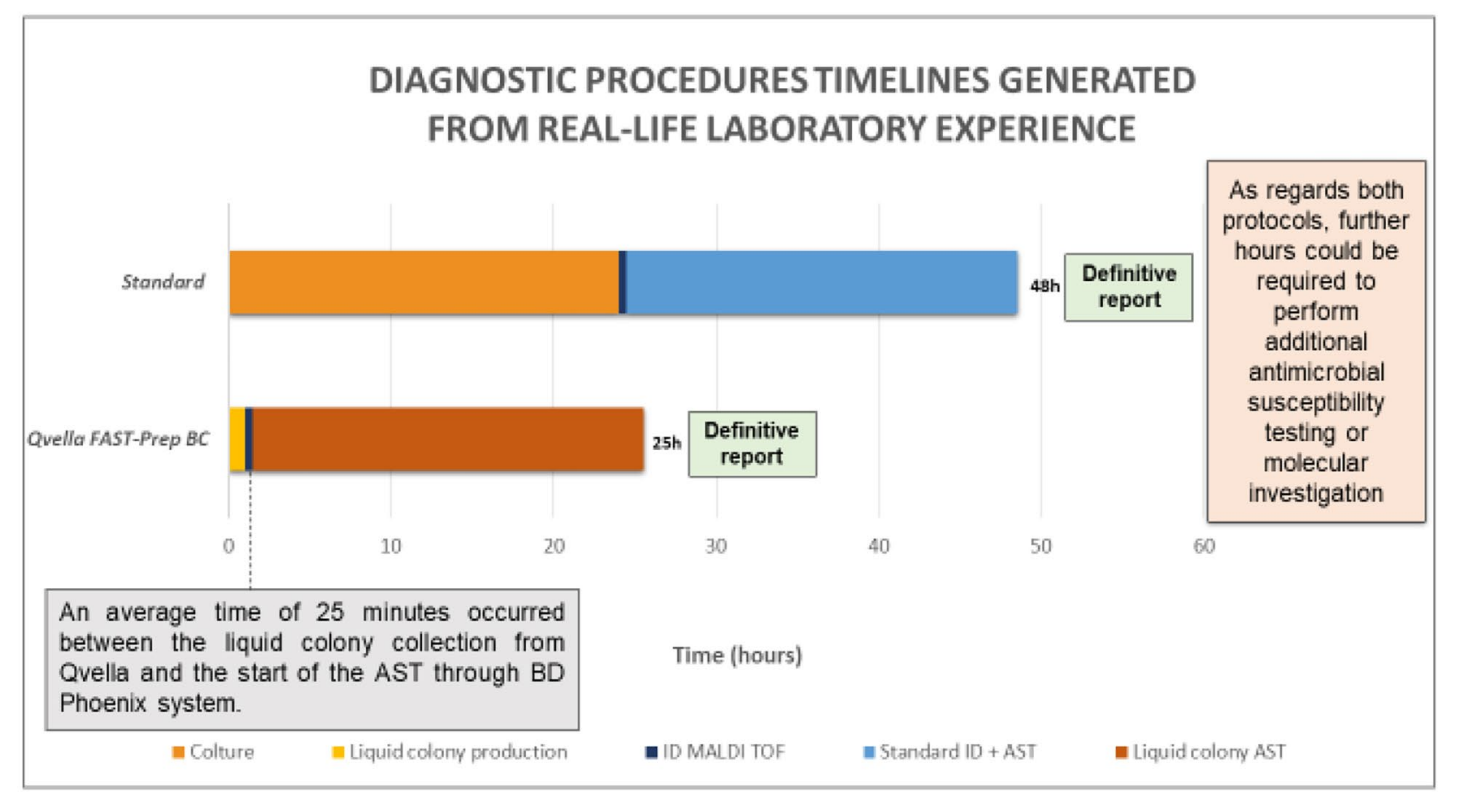
Comparison between standard procedure and the fast protocol for sepsis diagnosis. Every procedure implies a time to positivity before processing blood samples

The Standard procedure TAT analysis reported an overnight incubation period of 18–24 h, followed by the ID of the microorganism (median 26 h after blood culture positivity) and AST (median 50 h after blood culture positivity). The fast protocol involving the Qvella FAST System provided a report within 24 h. This analysis is graphically summarized in Graph 2.

A median time of 30 min was registered between the blood bottles extraction from the incubator and the execution of a Gram stain. The minimum and the maximum time observed for this interval were respectively 20 and 35 min. The median time between the interpretation of the Gram stain and the application of the FAST System was 30 min (with a minimum time of 15 min and a maximum time of 40 min). After the conversion in liquid colonies (30–40 min as a standard automated time), the MALDI ID was performed, requiring a short median interval of 25 min (with a minimum time of 20 min and a maximum time of 30 min). Overall, the total median turnaround time from blood culture positivity to ID was 2 h (with a minimum time of 1 h and 40 min and a maximum time of 2 h and 30 min). A 0.5 MacFarland suspension was then created from the LC and loaded in the Phoenix AST System to receive in the next morning a report. The median AST TAT was 26 h, with a minimum time of 24 h and a maximum time of 26 h.

## Discussion

This study aimed to evaluate the performance of the LC produced directly from positive blood samples by the Qvella FAST system in rapid ID and AST processes. ID and AST results from the LC was compared to the standard of care (SOC) using colonies obtained by subculture solid agar subculture. We also compared the ID and AST turnaround times for the FAST System workflow to standard processes.

Positive ID was recorded from the LC for all the examined samples and all IDs were concordant with results obtained with standard processes. The 100% correct ID rate is superior to previous studies evaluating MALDI ToF ID from the LC, in which a small percentage of samples resulted in either no ID or a discordant ID [14–17]. Our optimal result was likely due in part to the advanced technology of the MALDI Biotyper® Sirius System (Bruker). Another factor accounting for the improved ID rates is the timely workflow for processing PBCs and testing the LC. There was a relatively short time (~ 30 min) from blood culture positivity to processing of PBCs with the FAST System, and a relatively short time (~ 30 min) from PBC processing and obtaining the LC to MALDI ToF.

The highest ID scores were reported for all PBCs containing Gram-negative bacteria and most PBCs containing Gram-positive bacteria. On the other hand, lower-quality ID scores were sometimes obtained from PBCs containing Gram-positive bacteria, especially *Corynebacteria* and yeasts. We can hypothesize that the complex cell wall ultrastructure of these microorganisms is the leading explanation for sub-optimal results. This difficulty that we faced can also compromise MALDI ToF ID from colonies grown on solid agar. We cannot exclude possible ID inaccuracies due to the lysis, purification, and centrifugation automated stages of the FAST System.

In conclusion, further studies should be planned to investigate possible improvements for ID from LC-containing yeast, *Corynebacterium* spp., Gram-positive and uncommon microorganisms.

With regard to the evaluation of AST using the LC, we documented 100% CA and EA rates compared to standard processes. Despite the high percentage of samples containing highly resistant organisms evaluated in this study, we observed no minor, major, or very major errors. The small percentage of 1-dilution MIC differences that we observed is likely due to the intrinsic variability of MIC determination with automated AST systems such as BD Phoenix. Our AST results improve performances upon prior studies [14–19], providing concordant data after an automated concentration procedure. Certainly, the high concordance values are potentially due to the timely workflow for PBC processing PBCs, preparing the 0.5 MacFarland suspension from the LC, and inoculating the LC into the Phoenix cartridge. These results suggest that AST performance with the LC can be optimized either by avoiding the overgrowth of organisms in the blood culture or by timely preparation of the 0.5 MacFarland suspension from the LC and initiating the Phoenix AST run.

Despite advances in diagnostic technology, pathogen detection and identification remains a critical information gap during the earliest hours after sepsis onset. The LC appears to be a reliable tool for obtaining a definitive microbiology report within 24 to 48 h after blood cultures are obtained. This consideration confirms its high value in a sepsis diagnostic workflow, mainly thanks to a notable reduction in turnaround time. In line with these conclusions, our evaluation of the LC obtained using Qvella’s FAST System and FAST PBC Prep cartridge reveals an optimal agreement with the standard methods, offering an extended spectrum of microbiological information to support the clinical management of sepsis patients. In relation to other advantages, the LC homogenizes the entire population of microbial cells growing in the blood culture, reflecting all possible aspects of the susceptibility profile. For instance, Ugaban et al. document that heteroresistance episodes could be better detected through a LC than colonies grown on solid agar, which is examined selectively [17]. Moreover, we documented a time saving of 24 h or more over culture-based methods with our FAST System workflow, leading to prompt clinical and therapeutical management of septic patients.

The potential microbiological applications of a liquid colony are presented in Graph 3. As a final consideration, it would be interesting to promote further studies about other possible applications of the LC, such as whole genome sequencing and molecular detection of resistance markers. In our study, purification and concentration of microbial cells from a PBC represented an essential improvement in the diagnostic workflow for time-dependent infections.

Consequently, its application could be integrated into laboratory workflows because of the promising results and the considerable impact on critical patient outcomes. In line with this proposal, it is vital now to focus microbiologists’ attention on the necessity of presuming the best way to promote this strategic tool introduction. It would be worthwhile to endorse personnel education about the appropriate use of the technology by microbiologists and its effectiveness on the clinicians’ managing pronouncement. We proposed preliminary data, but extended clinical trials should be planned to verify and look at the possible applications in many other diagnostic tools. The most important purpose of a rapid sepsis diagnosis is to record essential information in a short time interval. In our opinion, the application of a liquid colony in a diagnostic workflow could contribute to satisfy this fundamental requirement.

## Data Availability

All data generated or analyzed during this study are included in this article.

## Declarations

### Author’s information

M.C. and G.M. are clinical microbiologists at the University Hospital Policlinico of Catania, via Santa Sofia 78, 95123, Catania, Italy. S.S. and G.S. are full professors of clinical microbiology at the University of Catania, Department of Biomedical and Biotechnological Sciences, Via Santa Sofia 97, 95123, Catania, Italy. They also hold a chief role at the University Hospital Policlinico of Catania, U.O.C. Laboratory Analysis Unit, Via Santa Sofia 78, 95123, Catania, Italy. M.P. contributes to research protocols and conceptualization studies at the University of Catania, Department of Biomedical and Biotechnological Sciences, Via Santa Sofia 97, 95123, Catania, Italy.
