# Correction: Evaluation of the liquid colony for identification and antimicrobial susceptibility testing directly from positive blood cultures

**DOI:** 10.1186/s12941-023-00629-4

**Published:** 2023-09-02

**Authors:** Calvo Maddalena, Migliorisi Giuseppe, Marianna Perez, Scalia Guido, Stefani Stefania

**Affiliations:** 1 U.O.C. Laboratory Analysis Unit, A.O.U. “Policlinico-San Marco”, Via S. Sofia 78, Catania, 95123 Italy; 2 Clinical Microbiology, Department of Biomedical and Biotechnological Sciences, Policlinico Hospital, University of Catania, Via Santa Sofia 97 Third floor, Est tower, Catania, 95124 Italy

Correction to: Annals of Clinical Microbiology and Antimicrobials (2023) 22:72

10.1186/s12941-023-00617-8

Following publication of the original article [1], the authors noticed some errors in the textual part.

These has now been corrected with this erratum.

1. Among the results section of the abstract, the sentence should read “100% ID identification concordance was observed across 47 Gram-negative bacteria, 42 Gram-positive bacteria and 11 yeast for the genus level” instead of “100% ID identification concordance was observed across 48 Gram-negative bacteria, 42 Gram-positive bacteria and 11 yeast for the genus level”.
2. Among the Discussion section, the sentence “The potential microbiological applications of a liquid colony are presented in Graph 3” should be removed from the text.
3. Among the Table 1, the *Acinetobacter baumannii* isolates number needs to become 8, accounting for a total number of 47 instead of 48 Gram-negatives.
